# Thinner bats to face hibernation as response to climate warming

**DOI:** 10.1038/s41598-024-52459-9

**Published:** 2024-01-24

**Authors:** Marc López-Roig, Eduard Piera, Jordi Serra-Cobo

**Affiliations:** 1 Departament de Biologia Evolutiva, Ecologia i Ciències Ambientals, Facultat de Biologia, Universitat de Barcelona, Av. Diagonal 643, 08028 Barcelona, Spain; 2 Institut de Recerca de Biodiversitat (IRBio), Facultat de Biologia, Universitat de Barcelona, Av. Diagonal 643, 08028 Barcelona, Spain; 3 Infraestructures.Cat (Generalitat de Catalunya), C/dels Vergós, 36-42, 08017 Barcelona, Spain

**Keywords:** Climate sciences, Ecology, Environmental sciences

## Abstract

One of the principal consequences of climate warming on hibernating mammals could be the loss of optimal conditions for hibernation. Although hibernating mammals, like bats, may be particularly vulnerable to climate warming due to a potential reduction of energy saving during the hibernation, there is a lack of knowledge regarding how they will be affected and how they will respond to this impact. Here, we examine the variation in the body condition of Schreiber’s bent-winged bat (*Miniopterus schreibersii*) to investigate changes in the optimization energy demand. Using a 20-year dataset (1998–2017), we analyse the temporal trends of body condition in three key stages of the hibernation period: onset and end of hibernation and early activity. Our results indicate that body condition at the onset and end of hibernation have decreased significantly over these 20 years. However, despite this lower body condition, the decrease of mass loss rate in the last decade (although not significant) indicate a greater saving of fat reserves. The significant increase in winter temperatures did not affect body condition or reserve depletion, instead, lower body condition was observed with a higher number of days below 0 °C. Unlike other hibernating bat species, the females had lower fat reserves than males in all three periods considered. This study indicates that hibernation energy requirements could be changing as an adaptation to a warmer climate and that hibernating bats can survive the winter by optimizing their lower accumulation of reserves.

## Introduction

The life cycles of species have evolved to maximize the fitness of individuals and the viability of populations by synchronizing key events seasonal (or phenologies) with weather optimal conditions and peaks of maximum resource availability^1^. Currently, there is evidence that global warming is advancing the phenology of many organisms. The activities of major events such as migration, reproduction or emergence of hibernation are occurring earlier in spring and summer than in past decades^2–4^. However, species, especially from different trophic levels, may have substantially different phenological responses to climate change, and this may alter the synchrony of ecological interactions and potentially affect wild populations and community persistence^5,6^.

The consequences of phenological advances have been indicated as beneficial in several fitness reproductive components (e.g. fecundity and clutch size) across a wide range of organisms^7–11^ but this is not always the case^10,12,13^. Despite our improved understanding of the various responses organisms face in the face of climate change, few studies have investigated how hibernating species will respond on optimization of energy reserves^14,15^.

Climate warming is modifying the hibernal period conditions by reducing the length and severity of winters^16^ and, as a consequence altering the phenology of hibernation. The alteration of winter weather conditions is already impacting hibernating organisms principally in temperate, alpine and polar environments, which they may spend up to half of their lives overwintering. It should be noted that organisms that may be particularly sensitive to climate warming are those that utilize torpor during hibernation like heterotherms mammal’s species^14^.

Hibernation is a physiological and behavioral adaptation that permits survival during seasonal periods of food shortage^17^. Over-winter survival of hibernating mammals depends primarily on the amount of energy reserves accumulated at the onset of hibernation, the rate at which the energy store is depleted during winter, and the length of the winter.

Hibernating mammals conserve energy throughout the multiday torpor, a period in which the metabolic rate and body temperature are greatly reduced. Prolonged and deep torpor will cause a greater energy saving but also involve in physiological costs^17^. To avoid these physiological consequences, torpor bouts are interrupted by short periods of arousals to achieve again a normothermic state. However, these arousals come with substantial costs (normally account for 80–90% of all energy utilized during the entire hibernation season)^18^. The balance between energetic benefits of torpor and costs of arousals cause individuals to optimize their expression of torpor during hibernation, and to avoid the use of deep torpor when possible^17^. However, this optimization of energy reserves can be altered if the ambient temperature is too high and does not allow the optimal torpor as ambient temperature dictates the degree of reduction in body temperature and metabolic rate during torpor^19^. An increase in the average of ambient temperature during autumn/winter may have direct negative effects on hibernators by shortening the duration of torpor bouts and increasing the number of arousal episodes. Therefore, one of the principal consequences of the increase autumn/winter temperatures associated with global warming will be the reduction the energetic savings necessary for the survival of individuals in winter^16^.

Bats constitute one of the most diverse and geographically dispersed groups of mammals and they have been considered especially vulnerable to several factors such as destruction of habitat, anthropogenic disturbance and climate change^20–22^. Climate warming impact has already been indicated in North American hibernating bats, increasing the total energy demand during winter and mortality risk, and as a consequence predicting a northward range expansion^14^. However, other temperate bats like a Mediterranean bat species could not respond in the same way to climate warming because the magnitude of impact can considerably differ between bat groups with different biogeographic patterns^23^. The current lack of data from wild hibernating populations limits our ability to predict how hibernating bat species will be affected by climate warming, especially in regions where the winters fade.

The main aim of this study was to investigate the effects of increasing temperatures during the autumn and winter period at a regional scale on the body condition of Schreiber’s bent-winged bat (*Miniopterus schreibersii*) colony located in the NE Iberian Peninsula. To achieve our aim, we used a 20-years period (1998–2017) of body condition data set in three key periods of hibernation phenology: onset and end of hibernation and early activity period. Additionally, we also employed the body mass depletion optimization as a “indicator” of energy expenditure during hibernation.

## Methods

### Study species

Schreiber’s bent-winged bat (*M. schreibersii*, Kuhl, 1817) is an insectivorous and cave-dwelling species of medium-sized bat (body mass of 10–18 g and forearm 45–48 mm) that almost invariably inhabits underground roosts, predominantly natural cavities and abandoned mine galleries. In these sites it can be locally abundant owing to its highly gregarious behavior, especially during its breeding and hibernation periods^24,25^. The roosting requirements of this species vary seasonally and condition strongly their annual cycle. After hibernation, females migrate to spring roosts, and later to maternity roosts where the births take place. Shortly afterwards when the juveniles are flying, they migrate to the autumn equinoctial roosts and finally to hibernation sites. The pattern of migration of males is similar to that of females, but they leave the hibernacula later and remain more mobile during the maternity season. They also arrive at the hibernacula later, possibly because they need more time to build up fat stores after the energetically costly mating season.

The hibernation sites are characterized by mild and stable temperatures (between 4 and 11.5 °C)^26^. Hibernation period in the Iberian Peninsula have been estimated in approximately two months, from mid-December to end-February^25,27^, although this period can vary depending on the inter annual weather conditions. Between these different roosts, *M. schreibersii* can perform relatively long displacements and movements of up to 250 km have been recorded in north east of Iberian Peninsula^28^. Consequently, it is considered a migratory species although at a regional level^29^.

### Study site and data collection

This study was carried out at Daví pothole, a cavity located in Sant Llorenç del Munt i l’Obac Natural Park (NE Iberian Peninsula, 41° 39ʹ N, 2° 1ʹ W; elevation 932 m). The vegetation is typically Mediterranean and consists of mixed forests of white pine, oaks, and holm oaks.

Extensive limestone karst is also present in this region.

Bats were captured over a 20-years period (from 1998–1999 to 2017–2018 winters) during the day inside the hibernacula with hand or when was not possible, with a long-handled butterfly net. Bats were captured from different sites of the cluster (from the edges to the center) and in several clusters when it was possible. To minimize disturbance to hibernating bats, the capture events occurred generally three times for the winter period (December, February and March) except for a 5-years period (2003–2007) in which we sampled two times (at December and February or March). In some years, the first captures were made in January (1st week) because of bad weather conditions that made access to the cavity difficult.

Bats were sexed, weighed to the nearest 0.01 g using a digital pocket scale (Pesola PPS200) and their forearm length was measured with a digital calliper (accuracy 0.01 mm). Bats captured for first time were marked with uniquely coded alloy forearm ring (Porzana Limited, East Sussex, UK) and the code was noted for all bats (including recaptured bats). All bats were released immediately inside the pothole after each capture session. Bat capture, handling and marking were authorized by a Government of Catalonia permit.

### Environmental variables

We used daily weather records from 1998 to 2018 taken at the closest reliable network Automatic Weather Stations (AWS, managed by the Servei Meteorològic de Catalunya) located in Sant Llorenç Savall (520 m.a.s.l.), at only 4 km away from the study site. The dataset had very few missing records (with only 29 instances), the most of which (26) were in 2004. To estimate these missing values, we used a complete data series from other meteorological AWS station (Caldes de Montbui), a locality approximately 20 km from our study area, with temperatures very closely correlated (*r*^2^ > 0.95).

To describe the climatic conditions affecting the hibernating bats in our study area, we considered three periods with differential impacts on bats body condition: (i) autumn (from November 1 to December 15), when bats accumulate fat reserves, (ii) winter (from December 15 to end-February), when bats hibernate, and (iii) early spring (March), when bats start the activity period. For each period, we used the weather variables: average minimum, mean and maximum daily temperatures, the numbers of days upper 10 °C and below 0 °C (winter severity) and winter length (calculated as the number of days that elapse between the first and the last three consecutive days with minimum temperatures below 0 °C).

### Body condition and mass loss calculation

Body condition (BC) was estimated using the method proposed by Peig and Green^30,31^ based on a standardized regression axis (SMA) instead of ordinary least squares (OLS) regression between individuals (Supplementary Appendix S1).

We focus on body condition (BC) as variation in mass can be largely attributed to pre-hibernation fattening and subsequent use of these reserves to fuel hibernation as bats generally do not feed during hibernation^32^. We considered three periods with a potential impact on BC: onset of hibernation when bats have high fat reserves (mid-December), end of hibernation (end-February) and mid-March as early activity period and migration. However, as body mass decreases during the winter, linear regression of body mass relative to the capture date (which indicated date of the survey in number of days since December 1) was used to calculate body mass corrected. The values obtained were used to calculate (using the scaled mass index describe above) the body condition corrected (BCc). We performed this correction for each sex and period (Supplementary Appendix S2). We selected December 15, February 28 and March 20 as reference dates for onset and end of hibernation and activity period respectively.

To analyze reserves depletion in bats between each period (from onset to end of hibernation and from end of hibernation to activity period), the mass loss (in grams and milligrams per day) was calculated as the difference between mean of BCc values obtained for each capture date (December, February and March) divided by number of days elapsed in each time interval (75 and 21 days respectively). Data obtained during the first week of March (2004 and 2007) were used both for the analyses of end hibernation and early activity periods.

### Data analyses

We tested the temporal trends in the six weather variables described in the environmental variables section. Linear regression models were used for minimum, mean and maximum daily temperatures, the numbers of days below 0 °C and upper 10 °C and winter length with year as explanatory variable (1998–2018).

We initially evaluate the temporal trends and differences between sexes in body condition corrected by capture date (BCc) for each of three periods considered (onset and end of hibernation and early activity). Linear mixed-effects models (LMMs) were fitted, using maximum likelihood, with individual as random effects to account for repeated measures. We started with global models, which included sex and year as factors and their interaction term. We also computed from the long-term data (1998–2017) one variable (decade) which were grouped 10-year intervals (1998–2007; 2008–2017). In this occasion, LMMs were also fitted to test the possible effect of sex and decade as fixed-effect factors and their interactions for each period.

To test the significant difference of inter-annual variation in mass loss between sexes we used analysis of covariance (ANCOVA) with sex and year as factors and their interaction term. Two-way ANOVA was used to evaluate the effect of sex and decade as factors using mass loss rate as response variable. We used error type II in these analysis because no significant interaction terms were obtained. In these analyses, comparisons between models using Akaike Information Criterion (AIC) and *anova* function was used to select the best model.

To test whether weather variables affect the body condition (BC) and mass loss rates we conducted linear regression analyses of environmental variables on these two response variables for each sex. Due to the high correlation between some of these variables, we carried out linear regressions separately for each variable and data points in the regressions were weighted by sample size (see Supplementary Appendix S3). In these analyses, we used the weather variables described in previous section: minimum, mean and maximum daily temperatures and the numbers of days below 0 °C and upper 10 °C for each time interval between data captures. For the three first variables, we calculate the average of temperatures for each time interval. In the case of onset of hibernation, BC was correlated with environmental data of the same variables considering the time interval from 30 days before to first data capture. Because in each interval time the weather conditions vary dependently of their duration and date, in this occasion, we use BC not corrected by capture date.

All statistical analyses were conducted using R version 4.2.2^33^. All LMMs were fitted with the statistical package ‘LME4’. We checked the residuals of all models for normality and homoscedasticity. All models were assessed visually (histograms and quantile/quantile plots) for normal distribution of residuals. The non-parametric Kruskal–Wallis test was used when homogeneity of variances was not meet. Results were considered significant if P ≤ 0.05 (*α* ≤ 0.05). All values are given as mean ± SE.

## Results

### Temporal trends in weather conditions

**Figure 1:**
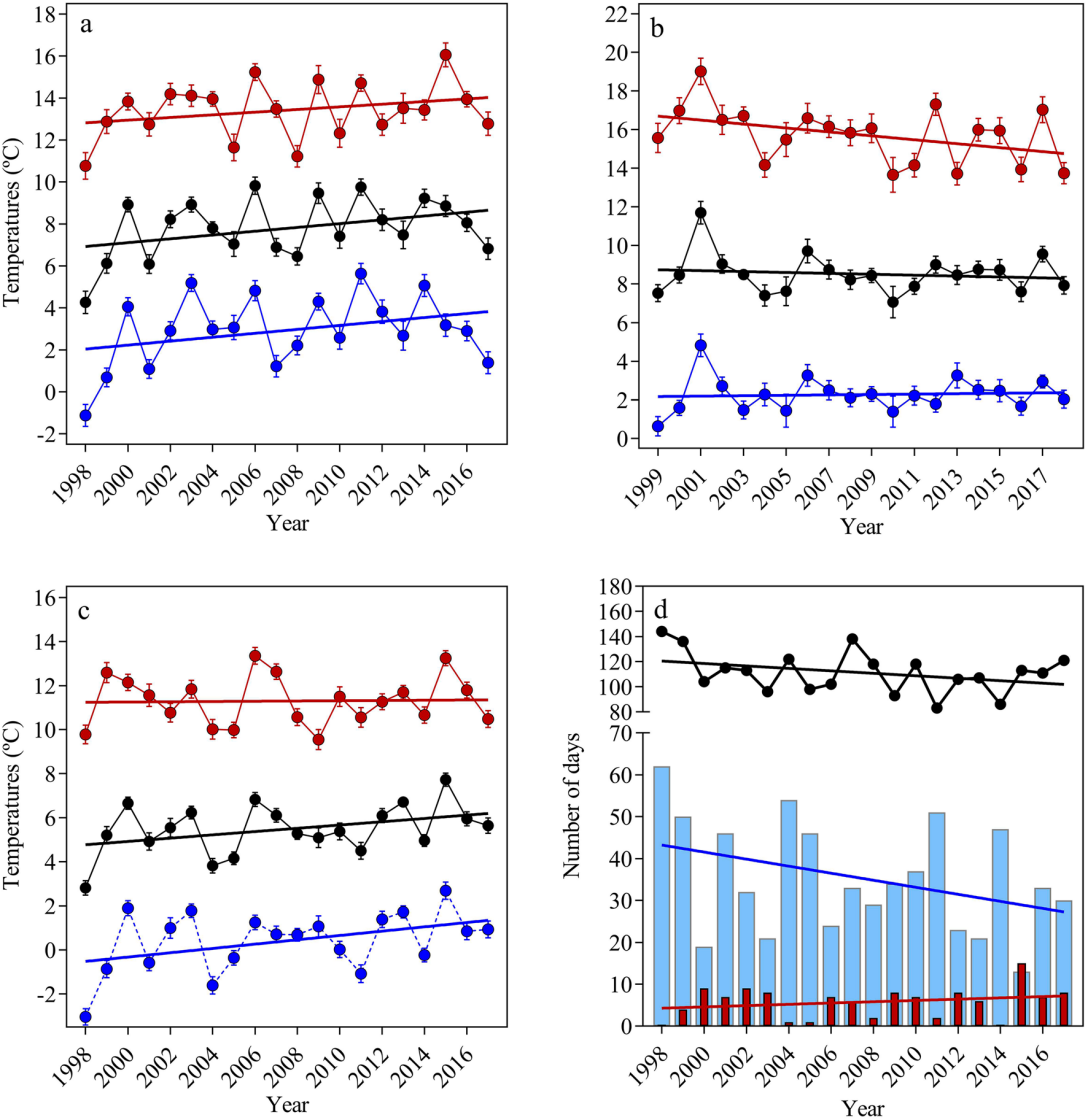
Mean, minimum and maximum temperatures in autumn (**a**), activity period (**b**) and winter (**c**) in our study area (mean ± SE). Number of days below 0 °C (blue bars) and upper 10 °C (red bars) and winter length (black points) are showed in graph (**d**). Years in x-axis for winter period (graphs **c,d**) indicate time interval between 15 December and end February for each year.

Autumn mean temperatures increased significantly between 1998 and 2017 in our study area (β = 0.092 ± 0.02, F_(1,898)_ = 22.41, P < 0.001, R^2^ = 0.024) (Fig. 1a). Maximum and minimum temperatures increased also significantly in autumn with an estimate of 0.066 °C per year (± 0.02, F_(1,898)_ = 9.19, P = 0.002, R^2^ = 0.010) and 0.095 °C per year (± 0.02, F_(1,898)_ = 20.01, P < 0.001, R^2^ = 0.022) respectively.

In winter period, the minimum and mean temperatures increased also significantly between 1998 and 2017 (β = 0.098 ± 0.01, F_(1,1518)_ = 41.15, P < 0.001, R^2^ = 0.026 and β = 0.075 ± 0.01, F_(1,1518)_ = 29.33, P < 0.001, R^2^ = 0.019, respectively). However, the same trend was not observed for maximum temperatures (F_(1,1518)_ = 0.13, P = 0.722) (Fig. 1c). Moreover, although there was an increase in the numbers of days with temperatures above 10 °C (β = 0.154 ± 0.15), no significant temporal trend was detected (F_(1,18)_ = 1.09, P = 0.309, R^2^ = 0.057) (Fig. 1d). Winter temperatures increased over time and, although winter severity (β =  − 0.840 ± 0.50, F_(1,18)_ = 2.84, P = 0.109, R^2^ = 0.136) and winter length (β = − 0.977 ± 0.61, F_(1,18)_ = 2.57, P = 0.126, R^2^ = 0.125) did not decrease significantly, negative trends suggest milder and shorter winters in this locality (Fig. 1d).

In the activity period, maximum temperatures decreased significantly with an estimate of 0.096 °C per year (± 0.03, F_(1,625)_ = 12.71, P < 0.001, R^2^ = 0.020) but no significant temporal trends were observed in mean and minimum temperatures (P > 0.30) (Fig. 1b).

### Temporal trends in body condition

We captured a total of 1589 bat individuals (781 females and 808 males). The average number of bats captured in each winter period was of 109 (± 19.87 SD) individuals and ranged from 78 (2006–2007) to 141 individuals (2017–2018).

#### Onset of hibernation

**Figure 2:**
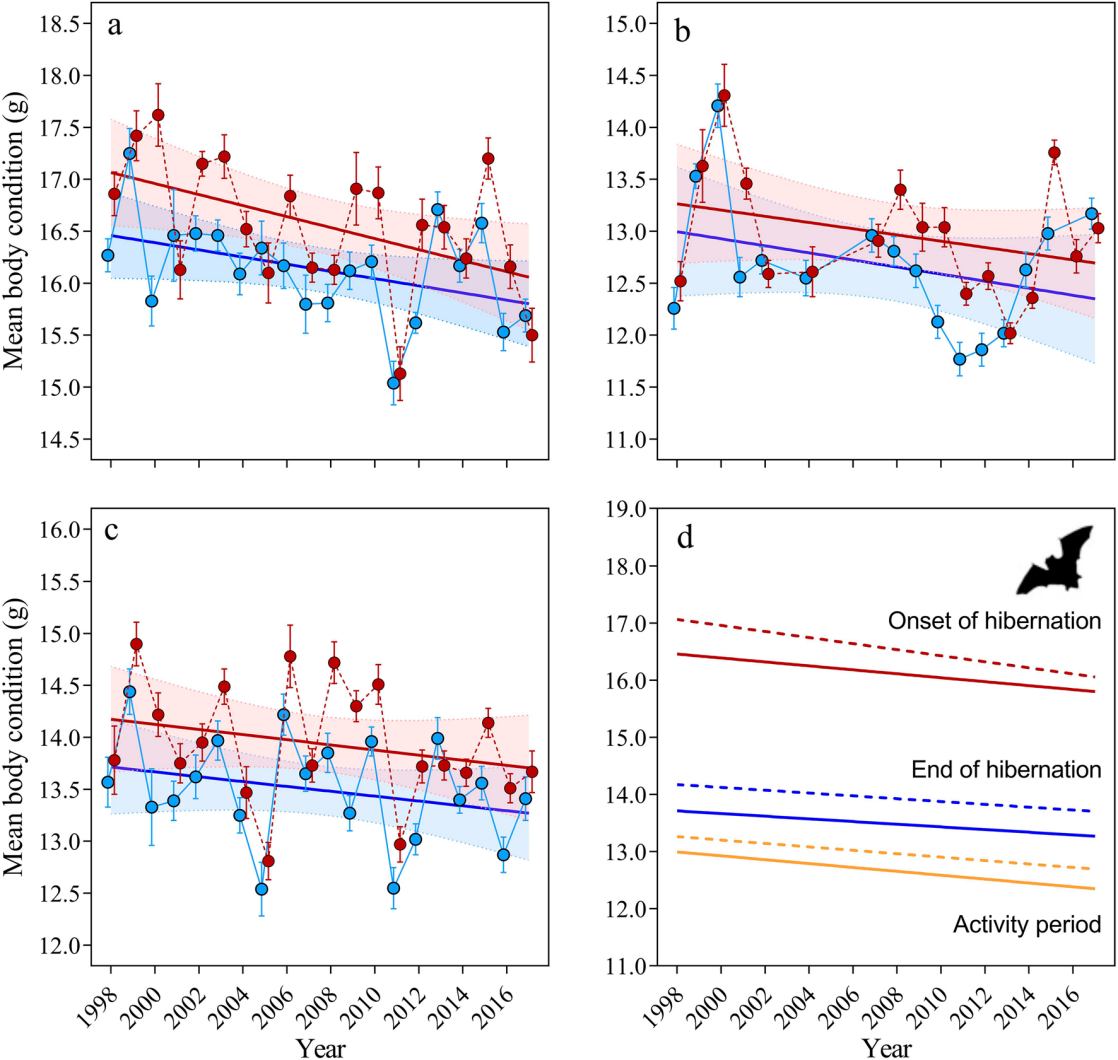
Body condition (BCc) (mean ± SE) between 1998 and 2017 in all three periods considered: (**a**) Onset and (**b**) activity period and (**c**) end of hibernation. Females (blue points) and males (red points). Graph (**d**) show temporal trends in all three stages: males (dashed line) and females (continuous line).

We used 803 bats (403 females and 400 males) to analyze the temporal trend in both sexes on body condition corrected (BCc) during this period. The model with and without interaction term between sex and year had a similar AIC (ΔAIC < 2) but reduced model was selected because no significant interaction was obtained (χ^2^_(1)_ = 3.21, P = 0.07). The model indicated a significant decrease between years (β =  − 0.044 ± 0.01, χ^2^_(1)_ = 47.29, P < 0.001). BCc trend of female was less pronounced (β =  − 0.033 ± 0.01, P < 0.001) than males (β =  − 0.055 ± 0.01, P < 0.001), which represents an average decrease of 0.66 g and 1.10 g respectively in this 20-year period. The BCc of females was also significantly lower (16.10 ± 0.05) than males (16.54 ± 0.06) (χ^2^_(1)_ = 37.46, P < 0.001) (Fig. 2a,d). When comparing by decade, we observed a significant higher BCc in the first years than last ones (χ^2^_(1)_ = 31.90, P < 0.001) (Table 1, Fig. 2a). We found no effect of interaction between sex and decade (P = 0.26).

**Table 1:** Body condition (BCc) by decade and total during all three periods considered.

Interval	Females		Males		Total	
	n	Mean ± SE	n	Mean ± SE	n	Mean ± SE
Onset of hibernation						
1998–2007	182	16.29 ± 0.07	197	16.79 ± 0.08	379	16.55 ± 0.05
2008–2017	221	15.94 ± 0.06	203	16.29 ± 0.08	424	16.11 ± 0.05
1998–2017	403	16.10 ± 0.05	400	16.54 ± 0.06	803	16.32 ± 0.04
End of hibernation						
1998–2007	158	13.64 ± 0.08	169	13.97 ± 0.09	327	13.81 ± 0.06
2008–2017	224	13.43 ± 0.06	257	13.83 ± 0.05	481	13.64 ± 0.04
1998–2017	382	13.52 ± 0.05	426	13.89 ± 0.05	808	13.71 ± 0.03
Early activity						
1998–2007	114	13.03 ± 0.09	105	13.09 ± 0.11	219	13.06 ± 0.07
2008–2017	187	12.58 ± 0.06	236	12.81 ± 0.05	423	12.71 ± 0.04
1998–2017	301	12.75 ± 0.05	341	12.90 ± 0.05	642	12.83 ± 0.04

*n* number of individuals.

#### End of hibernation

808 bats were considered (382 females and 426 males) in this period. The best model included the sex and year fixed effects without interaction. This model indicated that the BCc decreased significantly between years (β =  − 0.026 ± 0.01, χ^2^_(1)_ = 18.33, P < 0.001) but temporal trend did not differ between sexes (males: β =  − 0.027 ± 0.01, P = 0.001; females: β =  − 0.025 ± 0.01, P = 0.005) with a similar decrease of 0.54 g in males and 0.50 g in females (Fig. 2c,d). In this period, BCc of females was also significantly lower (13.52 ± 0.05) than males (13.89 ± 0.05) (χ^2^_(1)_ = 27.81, P < 0.001). Furthermore, mean BCc varied significantly between decades (χ^2^_(1)_ = 4.79, P = 0.029) showing a lower BCc in the last years (Table 1, Fig. 2c).

#### Activity period

During this period, 642 bats (301 females and 341 males) were captured. The model selection showed two models with ΔAIC < 2 but like previous case we selected as the best model as the one without interaction term because it wasn’t significant (χ^2^_(1)_ = 0.66, P = 0.415). The model indicated that females (12.75 ± 0.05) start the activity period in lowest BCc than males (12.90 ± 0.05) (χ^2^_(1)_ = 5.42, P = 0.020). This model also indicated a significant temporal trend decline on BCc (β =  − 0.025 ± 0.01, χ^2^_(1)_ = 17.55, P < 0.001) from 1999 to 2018 (Table 1, Fig. 2b,d). Similar temporal trends were observed in mean BCc for both sexes (females: β =  − 0.031 ± 0.01, P < 0.001; males: β =  − 0.021 ± 0.01, P = 0.015) with a decreasing of 0.62 g in females and 0.42 g in males (Fig. 2b). Significant higher BCc were obtained in first than in last decade (χ^2^_(1)_ = 22.15, P < 0.001) (Table 1, Fig. 2b).

### Temporal trends in mass loss

Mass loss rates differed between two periods (Dec–Feb: 35.07 mg ± 0.97; Feb–March: 43.00 mg ± 4.63) (χ^2^_(1)_ = 4.60, P = 0.032) indicating higher reserves saving in the first period. Mass loss rates between sexes were similar in both periods (P = 0.568).

**Figure 3:**
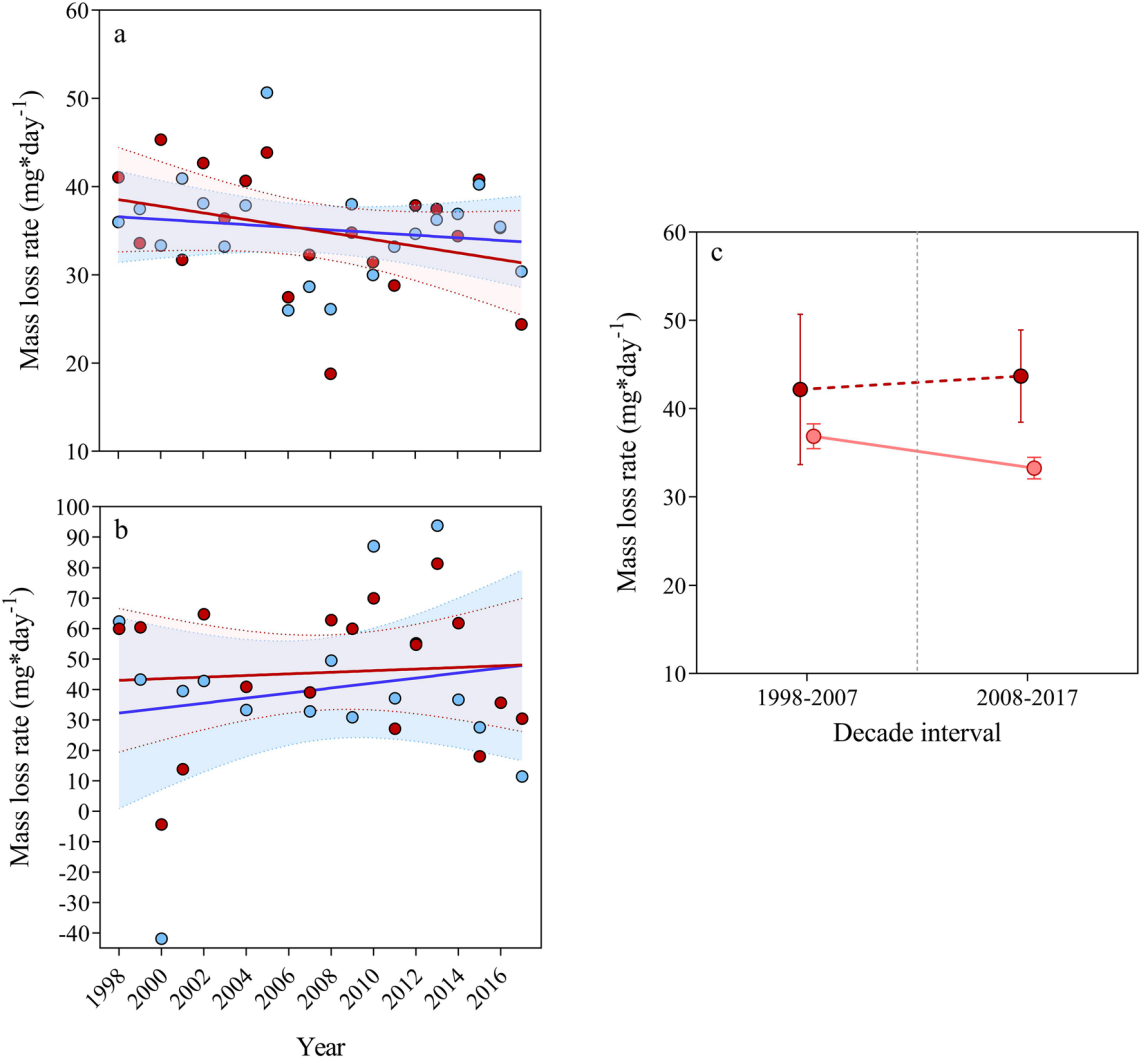
Mass loss rate from 1998 to 2017 between onset and end hibernation (**a**) and from end hibernation to early activity (**b**). Females (blue points) and males (red points). (**c**) Mass loss rate (mean ± SE) by decade intervals between onset-end of hibernation (continuous line) and end of hibernation to early activity (dashed line).

In hibernation period, mass loss followed a decreasing temporal trend being not significantly different between years (β =  − 0.263 ± 0.16, F_(1,38)_ = 2.53, P = 0.120) and also between sexes (F_(1,38)_ = 0.012, P = 0.912). When comparing decades we obtained a lower mass loss rate in the last decade (1998–2007: 36.86 mg ± 1.41; 2008–2017: 33.27 mg ± 1.24) (Table 2, Fig. 3a,c) although it was significant at 90% confident interval (F_(1,38)_ = 3.66, P = 0.063).

**Table 2:** Mass loss (g/mg day^−1^) by decade and total (mean ± SE) between two periods.

Period	Females	Males	Total
Onset-end hibernation	Total (g)		
1998–2007	2.72 ± 0.16	2.81 ± 0.14	2.76 ± 0.11
2008–2017	2.56 ± 0.10	2.43 ± 0.16	2.50 ± 0.09
1998–2017	2.64 ± 0.09	2.62 ± 0.11	2.63 ± 0.07

	Rate (mg day^−1^)		
1998–2007	36.23 ± 2.15	37.51 ± 1.91	36.87 ± 1.41
2008–2017	34.13 ± 1.35	32.41 ± 2.13	33.27 ± 1.24
1998–2017	35.18 ± 1.26	34.96 ± 1.51	35.07 ± 0.97

End hibernation-activity	Total (g)		
1998–2007	0.74 ± 0.26	1.15 ± 0.15	0.89 ± 0.18
2008–2017	0.98 ± 0.16	0.88 ± 0.15	0.92 ± 0.11
1998–2017	0.84 ± 0.16	0.96 ± 0.12	0.90 ± 0.10

	Rate (mg day^−1^)		
1998–2007	35.08 ± 12.30	54.95 ± 7.38	42.18 ± 8.53
2008–2017	46.60 ± 7.60	41.86 ± 7.19	43.61 ± 5.23
1998–2017	40.12 ± 7.59	45.71 ± 5.60	43.00 ± 4.63

In activity period, the temporal trend of mass loss rates was not significantly different between years (β = 0.547 ± 0.77, F_(1,31)_ = 0.50, P = 0.485) and also between sexes (F_(1,31)_ = 0.36, P = 0.554). Significant differences were not obtained also between decade (F_(1,31)_ = 0.02, P = 0.881) indicating similar mass loss rates between decades (1998–2007: 42.18 mg ± 8.53; 2008–2017: 43.61 mg ± 5.23) (Table 2, Fig. 3b,c).

### Weather effects on body condition and mass loss

There was no evidence that minimum, mean and maximum temperatures in autumn influenced the BC in any sex at the onset of hibernation (Supplementary Appendix S3). Number of days above 10 °C had significant effect on BC only in males (β = 0.108 ± 0.04, P = 0.023). However, the number of days below 0 °C for the same period had not a significant effect.

**Figure 4:**
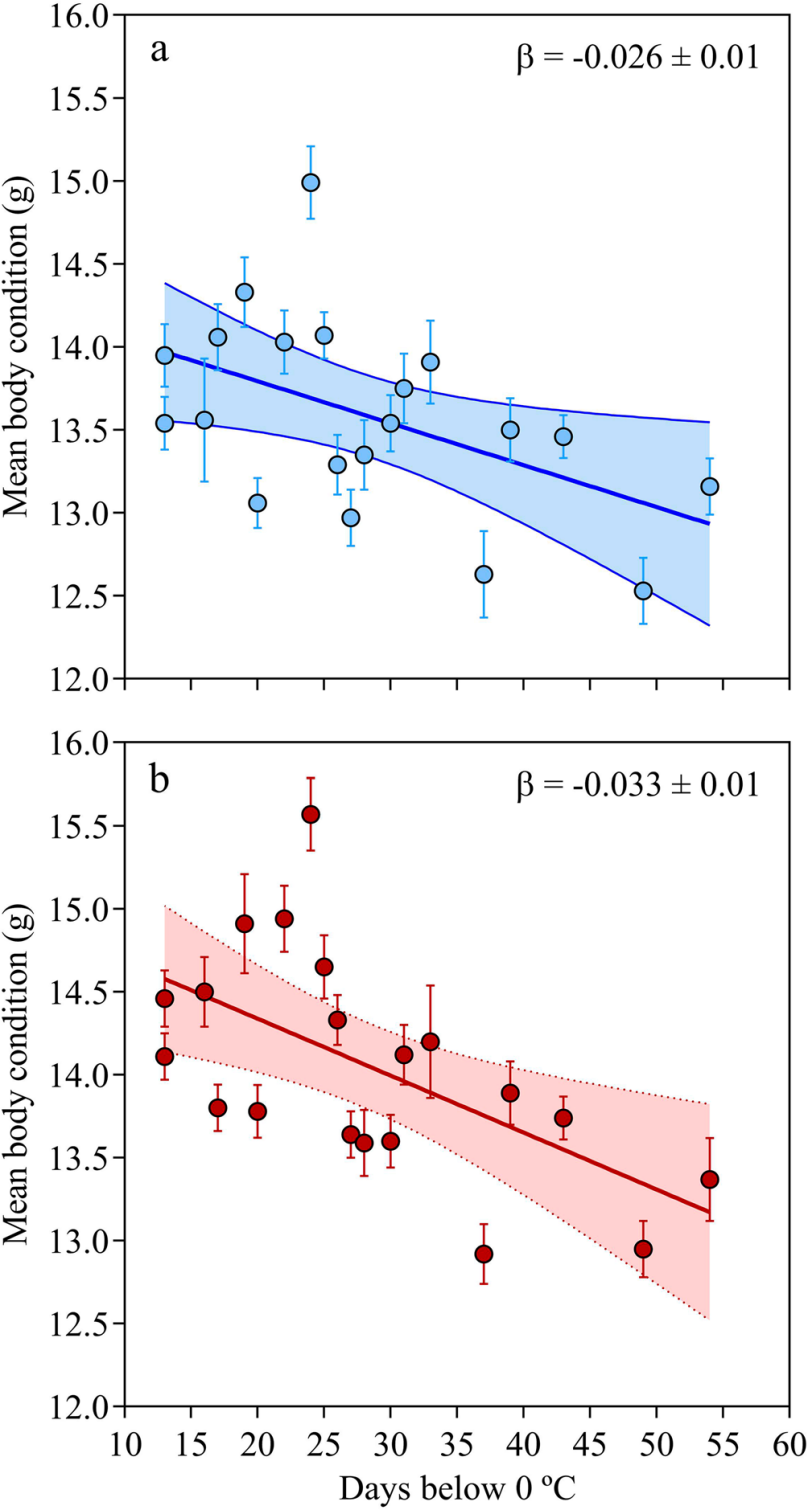
Relationship body condition (BC) (mean ± SE) at the end hibernation with respect to days below 0 °C in females (**a**) and males (**b**).

BC at end of hibernation also was not influenced by any three variables of the winter temperatures. However, winter severity (number of days below 0 °C) had a negative significant effect on BC in both sexes (females: β =  − 0.026 ± 0.01, P = 0.017; males: β =  − 0.033 ± 0.01, P = 0.007) (Fig. 4). None weather variables tested in activity period had a significant effect on BC by any sex (Supplementary Appendix S3).

There was also no evidence that weather variables tested influenced the mass loss at end of hibernation in any sex. Only the number of days upper 10 °C (β =  − 2.050 ± 0.91, P = 0.042) was significantly correlated with mass loss rates (BCL) of males in activity period (Supplementary Appendix S3).

## Discussion

The hibernation phenology of Schreiber’s bent-winged bat has changed significantly over the last 20 years decreasing the body condition of both sexes during winter period. Increased ambient temperatures may cause torpor bouts less efficient and increase energy of expenditure and as consequence a decrease in bat body condition. However, the bats that hibernate in our study area do not experience an increase in their spending on fat reserves as a consequence of local rising winter temperatures. A decreasing temporal trend of fat reserves depletion over the years and a lower mass loss rate in the last decade (although not significant) suggest that bats made a good optimization of their lower energy reserves accumulated in autumn. Our findings also provide evidence that bats start the early activity period and migration in spring with a lower body condition of both sexes than in previous years.

The decrease in body condition at the onset of hibernation implicates lower fat accumulation in autumn and less energy reserves to over-winter survival. Although warmer climatic conditions in autumn could favor a major insect availability and opportunity of feeding, the hibernating bats in our study area do not accumulate higher fat reserves before hibernation. The better body condition in males in autumn when the number of days above 10 °C increased, suggest that bats could feed punctually when environmental conditions are optimal^34,35^ minimizing their exposure front predators^36^. Sexual differences could be due to males arriving later than females in hibernation roost and they have higher energy requirements because of higher costs in mating season^37^.

The increase in minimum and mean temperatures in the winter period and a negative trend in the number of days below 0 °C indicate shorter and less severity winters. Autumn accumulation reserves should be larger in situations where winter energy requirements are higher because of long and hard winters^17^. Our results suggest that hibernation energy requirements could be shifting towards adaptation to a warmer climate and as consequence the hibernating bats accumulates fewer fat reserves to face ever shorter and milder winter periods.

The results also provide evidence that body condition of *M. schreibersii* at end of hibernation has been decreasing over the last two decades. Despite increases in winter temperatures, the body condition was not influenced by ambient temperatures suggesting that external weather conditions are buffered in the cave. The hibernacula temperatures are warmer than other sites in the pothole (~ 7 °C) and fluctuate slightly (± 1.9 °C) throughout winter (Unpublished data). In this sense, the cave offers a range of different microclimates that allows bats to select optimal temperatures and to minimize their energy expenditure, in such a way that individuals with lower body condition select colder places to energy saving^38^. Severe cold waves can cause a decrease more accused of the internal temperatures even in deeper chambers. This fact could increase the energy expenditure due to higher torpor cost associated with extremely colder temperatures. The negative relationship between body condition and an increase of days below 0 °C in both sexes suggest that lower temperatures in hibernacula could affect energy conservation. Although *M. schreibersii* tolerates ambient temperatures at least as low as 4 °C^26^, prolonged exposure to temperatures < 7 °C could lead to additional costs of thermoregulation similar to European free-tailed bat (*Tadarida teniotis*), another bat species of subtropical origin^39^.

Our results also indicate that both sexes begin the period of activity with worse body condition than in previous years, and besides, body condition of females were significantly thinner than males. This finding is very relevant because sex and body condition (or energy reserves) have been indicated to affect spring emergence phenology^40,41^. Norquay and Willis and Czenze and Willis predicted that female bats should emerge from hibernation earlier than males to initiate reproduction as early in the season as possible while the males should remain in hibernation longer than females and use their stored energy to reduce predation risk. However, an opposite phenological pattern has been observed in Iberian Peninsula^28,24^, in which females move later to equinoctials shelters. The body condition could govern such behavior by causing individuals with better body condition to leave the shelter earlier than those with worse body condition in order to recover fat reserves before initiating the migration. However, a poorer body condition compared to previous year could have important ecological implications especially in female bats as lower energy reserves could compromise the survival and reproduction success. In cold regions, where the winter duration is longer that in temperate regions, migration and gestation in spring often start shortly after hibernation^42,43^. Therefore, emerging from hibernation with a larger energy reserve may confer a reproductive advantage over individuals with small energy reserves^44,45^. However, spring weather in Mediterranean region are mild and insect activity is present, unlike higher northern latitudes where weather in spring are cold and food can be still unavailable. In this sense, the worse body condition in females in this period could have a minor impact as the time elapsed between end hibernation and start of parturition is long enough to allow individuals to recover their optimal body condition and to guarantee the energy requirements of reproduction.

Previous studies have demonstrated that energy reserves and sex influence patterns of torpor, arousal, and energy expenditure during hibernation^17,46^. According to the torpor optimization hypothesis^17^, we found a slight increase on energy savings in the last decade suggesting that hibernating bats with lower body condition (and fat reserves) exhibit higher energy conservation.

However, our findings indicate that energy savings did not differ between sexes in neither of the two periods considered and therefore don’t support the prediction of the thrifty female hypothesis^46^ where females should maximize energy savings because fat reserves are important to initiate pregnancy in spring. In our study area, females showed a worse body condition than males in all three periods considered contrary to previous studies in North American hibernating bat species^47^. In this regard, our results do not agree with the torpor optimization hypothesis, where males should expend more energy during hibernation than females. Other studies also indicated that lower temperatures promote longer and deeper torpor bouts and as a consequence lower energy expenditure^16,48^. Mass loss rate during hibernation was higher in *M. schreibersii* (35 mg/day) than in other temperate bats like little brown bat (*Myotis lucifugus*) (11 mg/day)^47^ indicating a lower energy saving. However, our study provides evidence that rising winter temperatures do not result in a greater increase of fat depletion and that the decreasing of energy expenditure observed in the last decade could indicate that higher energy saving is as result of lower body condition of individuals. As mentioned above, ambient temperatures can be buffered inside cave, not affecting the reserves depletion rate due to an increase in arousals (with a high energetic cost). The higher energy conservation observed in *Myotis lucifugus* could be due to differences in thermal preferences (lower optimal winter temperatures) and a longer and severe winters in relation to winters (milder and shorter) in our study area. Optimization of energetic saving during hibernation could be conditioned by differences in the energy used for torpor, euthermia and arousals but also by aspects behavioral such as microclimate selection^38^ and cluster formation. In this sense, *M. schreibersii* is a gregarious species that form big clusters which could reduce the reserves depletion decreasing energy cost of arousals^49^.

*M. schreibersii* has been considered a bat species with higher score of risk factors that could be impacted by climate change^22^. However, Mediterranean bat species would be less affected by climate change because they are already adapted to warm conditions^23^. Our findings indicate for first time that the increase in winter temperatures in the Mediterranean region might not have a negative impact on hibernating bat species, especially those species adapted to warmer climates. In addition, rising temperatures provide the opportunity to colonize new regions that were previously unfavorable, and thus a northward expansion of current distributions is predicted in response to a warmer climate. However, an increase of temperatures can also have negative effect modifying the microclimate of caves and become a non-optimal refuge for hibernation. This impact could cause seasonal changes in the use of refuges and even modify the migratory routes known so far. The roosts loss, especially the hibernation shelters, may pose an additional treat to bats survival because the availability of roosts is one of the most limiting resources for bats^50^.

Another impact that bats will face with future climate change is whether the abundance of insects will match with their ecological requirements. The early appearance of several insect species has been indicated in response to warming climate^51^, which would have a positive effect on food availability for bats that advance their emergence of hibernation. This fact would have a positive impact as it would help the *M. schreibersii* in poor body condition to recover its fat reserves to face the migration and reproduction event. However, unpredictability of spring weather such as sudden cold waves could reduce chance of survival and reproductive success in bats with very few fat reserves to face a period without food availability.

A greater understanding of the changes that will take place during hibernation as a consequence of climate change will provide useful information for official environmental agencies to ensure the protection of key refuges that host hibernating bat colonies.

### Ethics statement

The study followed the ethical recommendations of the Spanish Legislation (Law 42/2007) and was approved by the Bioethics committee of the University of Barcelona. Bat sampling was authorized by the Department of Climate Action, Food and Rural Agenda of the Generalitat de Catalunya and the permission of the Natural Park of Sant Llorenç del Munt and Serra de l’Obac. All experiments were performed avoiding pain and in such a way as to minimize animal discomfort. The study was conducted in accordance with the ARRIVE guidelines (https://arriveguidelines.org).

### Supplementary Information

Supplementary Information.

## Data Availability

The datasets used and/or analyzed during the current study available from the corresponding author on reasonable request.
