# Supplementary material for: Thinner bats to face hibernation as response to climate warming
Source: Sci Rep. 2024 Jan 24;14:2117. doi: 10.1038/s41598-024-52459-9 (PMC10808443; doi:10.1038/s41598-024-52459-9)
Supplement: Supplementary file 1 — Supplementary Information. [file 41598_2024_52459_MOESM1_ESM.docx]

**SUPPLEMENTARY INFORMATION**

**APPENDIX S1**

**Body condition calculation**

Stepwise procedure to compute the scaled mass index (M) of body condition from body mass (M_i_) and a linear body measurement (L_i_) was used following Peig & Green (2009).

As the variation body mass of bats varied greatly throughout year, we restricted the data set in the period where the bats were in lower body mass (from 15 to 24 March). For this analysis, we used 134 individuals (60 females and 74 males). We choose the forearm as the linear body measurement and we calculate the mean of forearm (L_0_) from all individuals in the data set.

We obtained these results:

L_0_ = 46 mm

Slope (b_SMA_): 3.30 (CI: 2.83 - 3.86)

R^2^ : 0.173

P-value < 0.001

Figure S2.1. Linear regression between log body mass and log forearm.


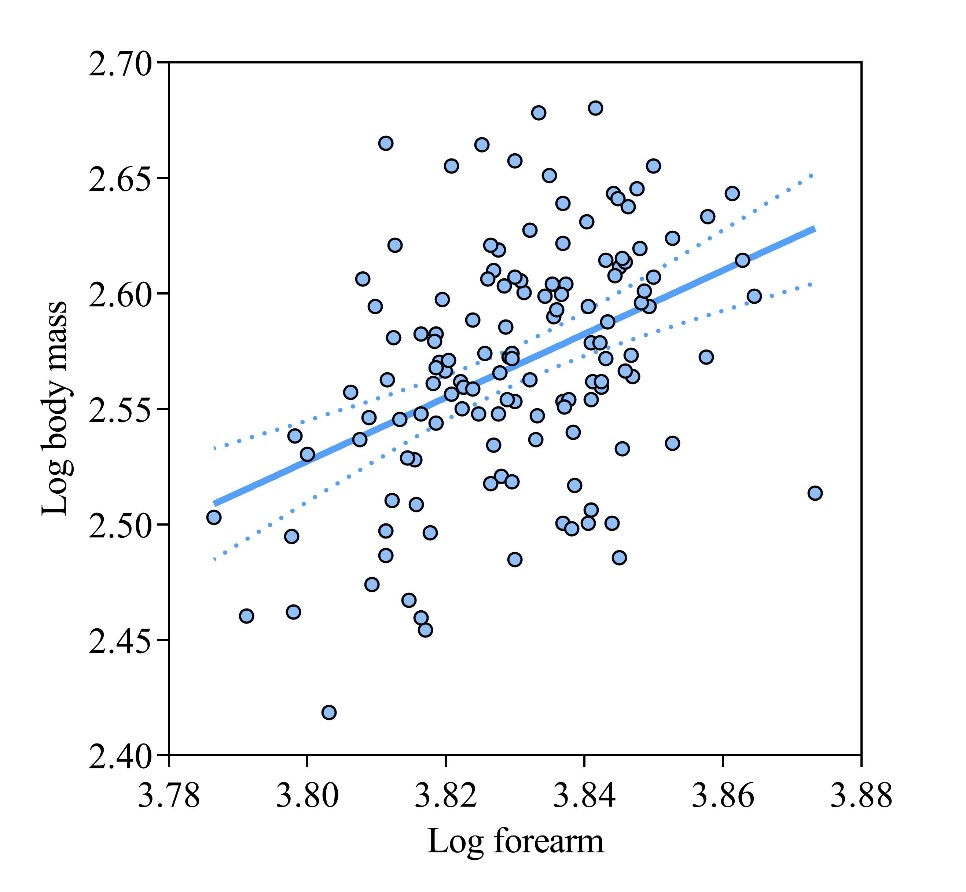


References

Peig, J., & Green, A. J. (2009). New perspectives for estimating body condition from mass/length data: the scaled mass index as an alternative method. Oikos, 118(12), 1883–1891.

**APPENDIX S2**

**Body condition correction**

As the body weight of bats decreased throughout winter, linear regressions between body weight and winter day by each sex were used to calculate the body weight corrected by capture date. The body weight corrected was obtained for the three periods considered (onset and end hibernation and activity period).

Hibernation period in the Iberian Peninsula have been estimated in approximately two months, from mid-December to end-February (Serra-Cobo, 1989 and 1998; Rodrigues & Palmeirim, 2008). For this reason, we grouped the data obtained between December-January as onset hibernation and between February and March (first days) as end-hibernation. Data obtained in mid-March were considered for activity period. Therefore, we selected December 15, February 28 and March 20 as reference data for onset and end of hibernation and activity period respectively.

References

[Serra-Cobo J. (1989). *Estudi de la biologia i ecologia de Miniopterus schreibersii*. PhD thesis. Universitat de Barcelona, 1000 p.](http://biodiver.bio.ub.es/biocat/servlet/biocat.BuscaDatUTMServlet?t5.1%7C@taxon@%25codi_e_orc=CHVMCHVE00009A%25codi_espe=CHVMCHVE00009A%25nivell=SP%7C%25c_bib=5953%25screenWidth=1280%25idioma=CAT%25cusu=1194355598718iKA)

Serra-Cobo, J., Sanz-Trullén, V., & Martinez-Rica, J. P. (1998). Migratory movements of *Miniopterus schreibersii* in the north-east of Spain. *Acta Theriologica*, 43, 271–283.

Rodrigues, L., & Palmeirim, J.M. (2008). Migratory behaviour of the Schreiber’s bat: when, where and why do cave bats migrate in a Mediterranean region? *Journal of Zoology*, 274(2), 116–125.

*Onset of hibernation*

Body mass decreased significantly throughout winter days in both sexes. Linear regressions by sexes are indicated in the figure S2.1. Males: (*χ^2^* = 29.45, df=1, *P* < 0.001). Females: (*χ^2^* = 56.38, df=1, *P* < 0.001).

Figure S2.1. Linear regression between body mass and winter day by sexes (females: blue circles; males: red circles) from bats captured in onset of hibernation. Line pointed indicates December 15 date.

**
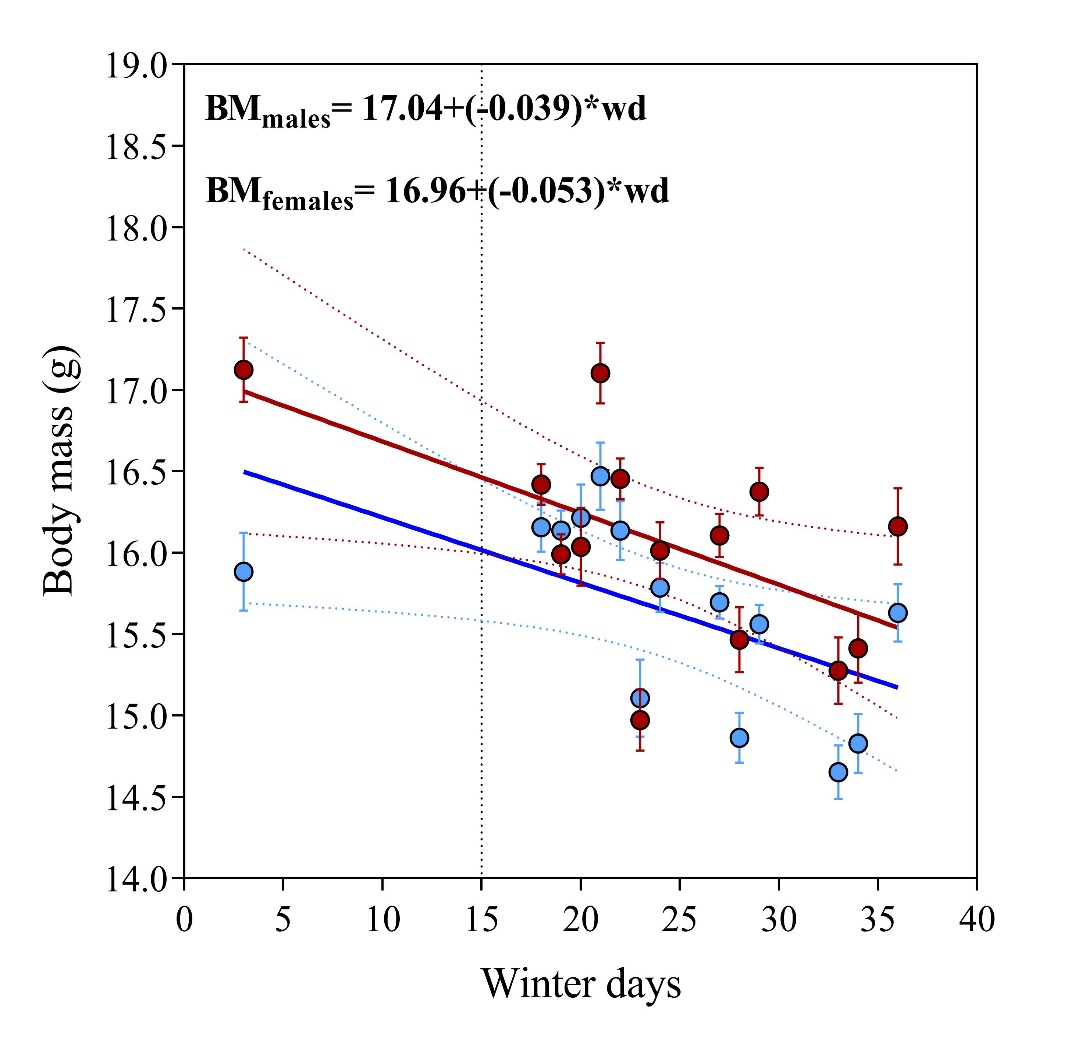
**

*End of hibernation*

Body mass decreased significantly throughout winter days in both sexes. Linear regressions by sexes are indicated in the figure S2.2. Males: (*χ^2^* = 13.93, df = 1, *P* < 0.001). Females: (*χ^2^* = 8.05, df = 1, *P* = 0.004).

Figure S2.2. Linear regression between body mass and winter day by sexes (females: blue circles; males: red circles) from bats captured in end of hibernation. Line pointed indicates February 28 date.


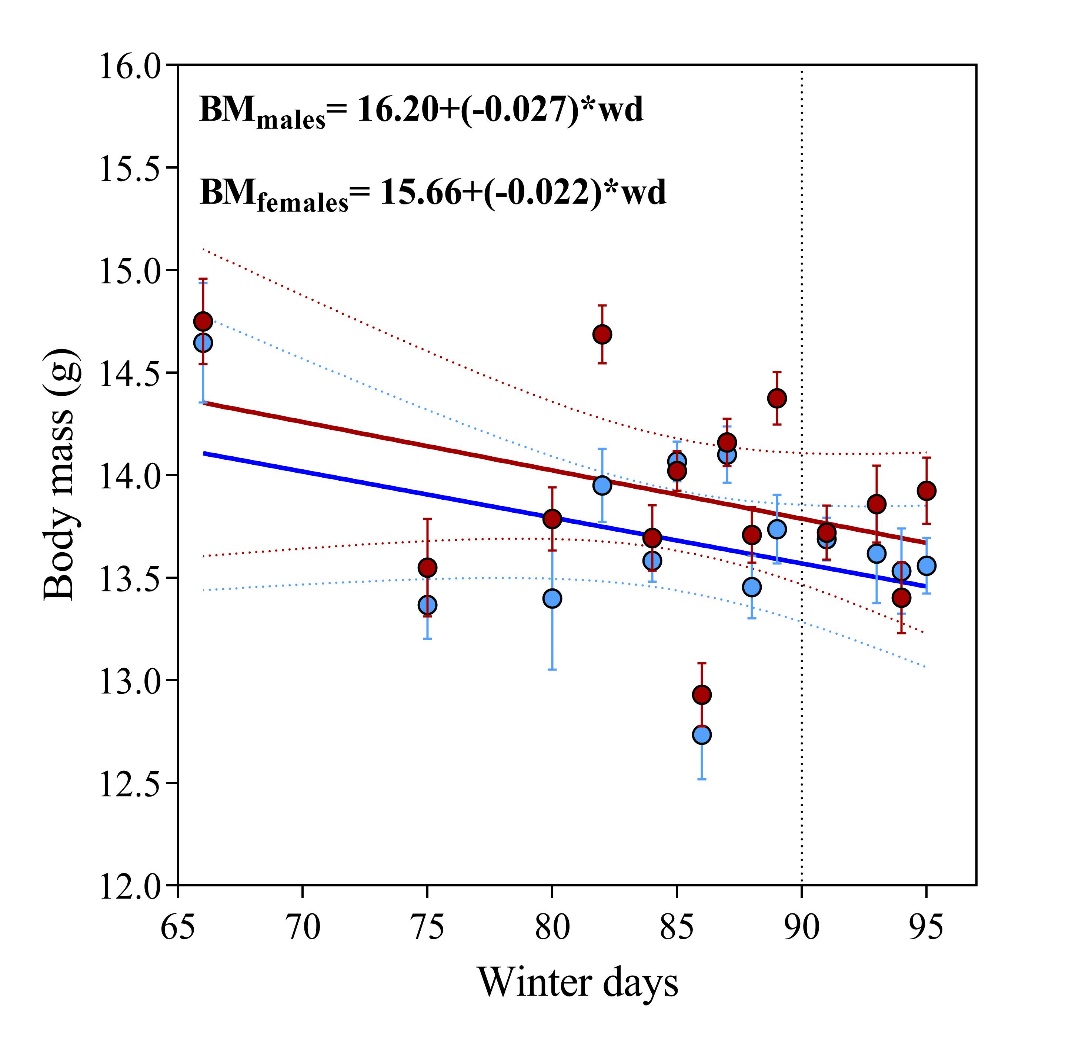


*Activity period*

Body mass decreased significantly throughout winter days in both sexes. Linear regressions by sexes are indicated in the figure S2.3. Males: (*χ^2^* = 61.67, df = 1, *P* < 0.001). Females: (*χ^2^* = 53.94, df = 1, *P* < 0.001).

Figure S2.3. Linear regression between body mass and winter day by sexes (females: blue circles; males: red circles) from bats captured in early activity. Line pointed indicates March 20 date.

**
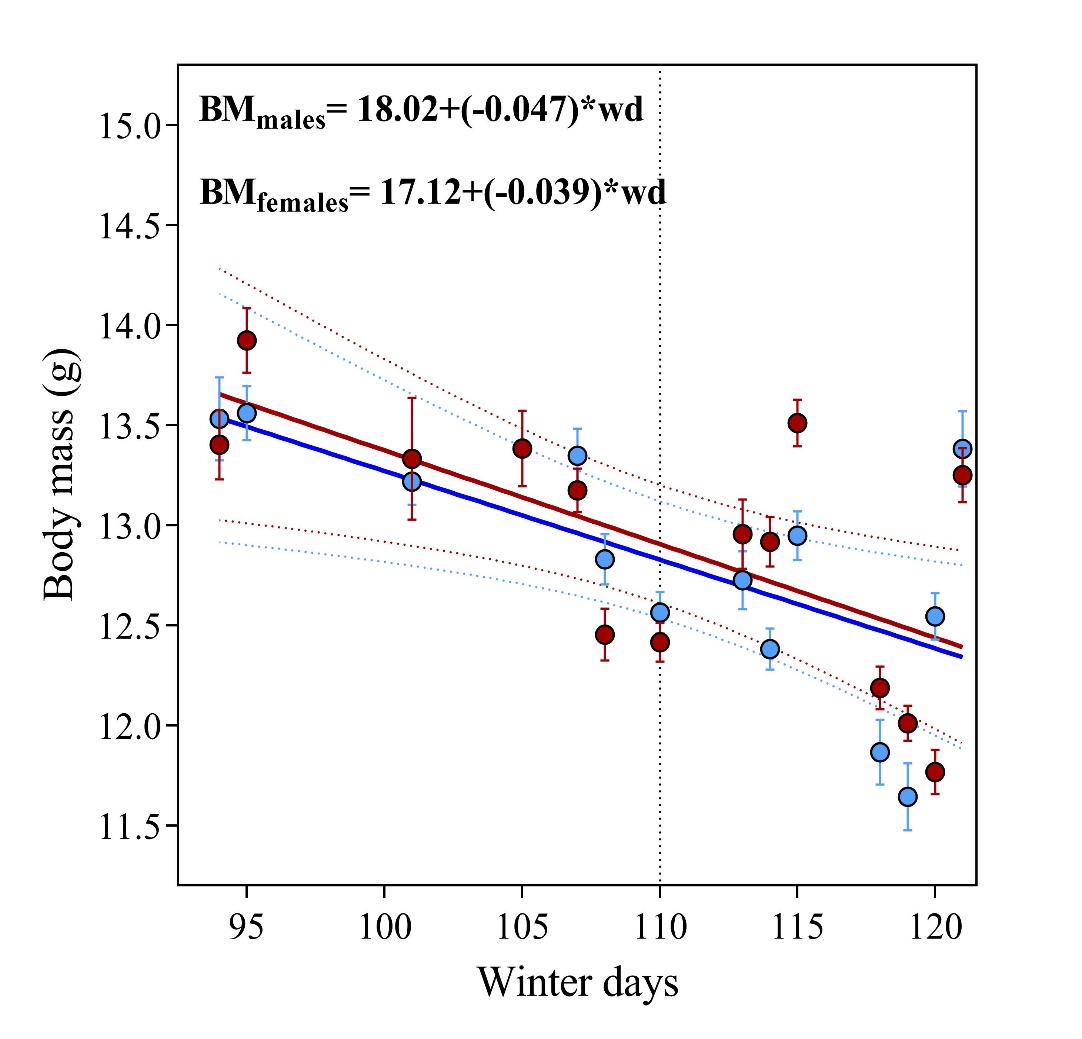
**

**APPENDIX S3**

## Tables of statistical results and correlations within climate factors

Factors tested against inter-annual variation in body condition and body condition loss rates for the Schreiber’s bat (*Miniopterus* *schreibersii*) in the study area. For all response variables, univariate linear regressions were used by sexes separately. All models were weighted by sample size: for the body condition regressions this comprised the number of animals sampled and for body condition loss rate we used the number of animals sampled in two occasions of sampling in each interval time.

Table S3.1. Pearson’s correlation coefficients between climate variables. Sample size for all variables in autumn and winter period is 20. For activity period, sample size is 15 for males and 14 for females.

|  | Minimum temperature | Mean temperature | Maximum temperature | Days below 0 ºC | Days upper 10 ºC |
| --- | --- | --- | --- | --- | --- |
| Minimum temperature | 1,000 | 0,873 | 0,544 | -0,756 | 0,640 |
| Mean temperature | 0,873 | 1,000 | 0,869 | -0,739 | 0,680 |
| Maximum temperature | 0,544 | 0,869 | 1,000 | -0,579 | 0,537 |
| Days below 0 ºC | -0,756 | -0,739 | -0,579 | 1,000 | -0,336 |
| Days upper 10 ºC | 0,640 | 0,680 | 0,537 | -0,336 | 1,000 |

Table S3.2. Statistical results of linear models of climate factors on body condition and mass loss rates. F-values < 0.05 are showed in bold and F-values < 0.1 in italic.

|  |  |  | **Onset hibernation** | | **End hibernation** | | **Activity period** | |
| --- | --- | --- | --- | --- | --- | --- | --- | --- |
| **Response variable** | **Variables** | **Sex** | **Slope** | **P-value** | **Slope** | **P-value** | **Slope** | **P-value** |
| Body condition (g) | Minimum temperature | females | -0,002 ± 0,07 | 0,978 | 0,252 ± 0,08 | 0,622 | 0,091 ± 0,17 | 0,594 |
|  |  | males | 0,081 ± 0,09 | 0,372 | 0,072 ± 0,10 | 0,479 | 0,110 ± 0,14 | 0,446 |
|  | Mean temperature | females | 0,008 ± 0,08 | 0,913 | 0,071 ± 0,10 | 0,474 | 0,151 ± 0,16 | 0,363 |
|  |  | males | 0,123 ± 0,10 | 0,215 | 0,081 ± 0,12 | 0,507 | 0,058 ± 0,14 | 0,690 |
|  | Maximum temperature | females | 0,014 ± 0,08 | 0,854 | 0,135 ± 0,10 | 0,200 | 0,137 ± 0,12 | 0,275 |
|  |  | males | 0,124 ± 0,10 | 0,217 | 0,125 ± 0,13 | 0,356 | -0,008 ± 0,11 | 0,941 |
|  | Days below 0 ºC | females | 0,012 ± 0,02 | 0,493 | **-0,026 ± 0,01** | **0,017** | -0,014 ± 0,04 | 0,736 |
|  |  | males | -0,010 ± 0,02 | 0,687 | **-0,033 ± 0,01** | **0,007** | -0,027 ± 0,03 | 0,432 |
|  | Days upper 10 ºC | females | 0,057 ± 0,04 | 0,141 | 0,011 ± 0,03 | 0,743 | 0,022 ± 0,04 | 0,628 |
|  |  | males | **0,108 ± 0,04** | **0,023** | 0,030 ± 0,03 | 0,384 | 0,017 ± 0,04 | 0,687 |
| Mass loss (mg/day) | Minimum temperature | females |  |  | -0,628 ± 0,98 | 0,530 | -4,709 ± 5,18 | 0,381 |
|  |  | males |  |  | -0,184 ± 1,21 | 0,881 | *-7,048 ± 3,60* | *0,072* |
|  | Mean temperature | females |  |  | -1,071 ± 1,15 | 0,362 | -5,334 ± 5,03 | 0,310 |
|  |  | males |  |  | -0,266 ± 1,43 | 0,855 | -6,338 ± 3,57 | 0,100 |
|  | Maximum temperature | females |  |  | -1,545 ± 1,26 | 0,235 | -2,597 ± 3,84 | 0,511 |
|  |  | males |  |  | -0,093 ± 1,61 | 0,954 | -2,810 ± 2,97 | 0,361 |
|  | Days below 0 ºC | females |  |  | 0,158 ± 0,14 | 0,264 | 0,935 ± 1,26 | 0,473 |
|  |  | males |  |  | 0,005 ± 0,16 | 0,978 | 1,518 ± 0,92 | 0,124 |
|  | Days upper 10 ºC | females |  |  | -0,242 ± 0,40 | 0,556 | -1,898 ± 1,35 | 0,186 |
|  |  | males |  |  | 0,310 ± 0,43 | 0,483 | **-2,050 ± 0,91** | **0,042** |
